# Genome-Wide Identification of mRNAs, lncRNAs, and Proteins, and Their Relationship With Sheep Fecundity

**DOI:** 10.3389/fgene.2021.750947

**Published:** 2022-02-08

**Authors:** Chunxin Wang, Yunhui Zhao, ZhiYu Yuan, Yujin Wu, Zhuo Zhao, Cuiling Wu, Jian Hou, Mingxin Zhang

**Affiliations:** ^1^ Institute of Animal Sciences, Jilin Academy of Agricultural Sciences, Changchun, China; ^2^ State Key Laboratory of Agrobiotechnology, China Agricultural University, Beijing, China

**Keywords:** sheep, multiple births, singleton, ovaries, multi-omics

## Abstract

The exploration of multiple birth-related genes has always been a significant focus in sheep breeding. This study aimed to find more genes and proteins related to the litter size in sheep. Ovarian specimens of Small Tail Han sheep (multiple births) and Xinji Fine Wool sheep (singleton) were collected during the natural estrus cycle. Transcriptome and proteome of ovarian specimens were analyzed. The transcriptome results showed that “steroid hormone biosynthesis” and “ovarian steroidogenesis” were significantly enriched, in which *HSD17B1* played an important role. The proteome data also confirmed that the differentially expressed proteins (DEPs) were enriched in the ovarian steroidogenesis pathway, and the CYP17A1 was the candidate DEP. Furthermore, lncRNA MSTRG.28645 was highly expressed in Small Tailed Han sheep but lowly expressed in Xinji fine wool sheep. In addition, MSTRG.28645, a hub gene in the co-expression network between mRNAs and lncRNAs, was selected as one of the candidate genes for subsequent verification. Expectedly, the overexpression and interference of *HSD17B1* and MSTRG.28645 showed a significant effect on hormone secretion in granulosa cells. Therefore, this study confirmed that *HSD17B1* and MSTRG.28645 might be potential genes related to the fecundity of sheep. It was concluded that both HSD17B1 and MSTRG.28645 were critical regulators in the secretion of hormones that affect the fecundity of the sheep.

## Introduction

Small Tail Han sheep is famous for its early maturity, perennial estrus, and polyembryony. The average litter size of excellent Small Tail Han sheep is 3.2 per parturition (Yuan et al., 2019). Xinji fine-wool sheep is a new breed of fine-wool sheep bred in China in 2003 and is characterized by single birth. Both Small Tail Han sheep and Xinji fine-wool sheep have stable genetic characteristics. However, the litter sizes of these two sheep are different. Sheep multiple births have always been one of the goals of sheep breeding. Understanding the molecular mechanism is essential for multiple births sheep breeding. Previous studies have shown that the prolificacy trait is quantitative and controlled by multiple genes (Tang et al., 2019; Yang et al., 2019; Esmaeili-Fard et al., 2021). Identification of genes associated with reproduction is essential for sheep breeding. Such genes can be introduced in breeding through marker-assisted selection whereby they can rapidly infuse superior genotypes in the breeding population (Oraon et al., 2016; Rout et al., 2018). It has been reported that the booroola fecundity gene (FecB) is an autosomal gene, which enhances ovulation rate through a codominant effect on litter size and partial dominance (Fogarty, 2009). Furthermore, the mutation in this gene has been revealed to be essential for ovulation rate. For example, polymorphisms in exon 2 of MTNR1A may regulate ewes’ reproductive seasonality and litter size by influencing gene expression (He et al., 2019). In addition, several genes related to fecundity, such as *KLF5*, *MYH15*, and *FecB*, have been identified in recent years (Miao et al., 2016a; Miao et al., 2016c; Nosrati et al., 2019). In addition, LncRNAs play an essential role in many life activities, including cell cycle regulation, cell differentiation, and cell epigenetic regulation (Arun et al., 2012; Abdelmohsen et al., 2013; Ghosal et al., 2013; Fatica and Bozzoni, 2014). They have been identified as essential regulators in the hypothalamic–pituitary–ovarian (HPO) axis associated with reproduction (Zheng et al., 2019). Miao et al. analyzed the ovaries of Small Tail Han and Dorset sheep and found that differentially expressed lncRNAs were significantly enriched in the oxytocin signaling pathway. It has been reported that methylation of lncRNAs might improve the reproduction of Small Tail Han sheep (Miao et al., 2016a; Miao et al., 2017). A study by Feng et al. (2018) identified five differentially expressed lncRNAs by analyzing the ovaries of Hu sheep with high and low reproduction rates. They found that lncRNAs in the ovaries of sheep have regulatory functions in reproduction. With the development of proteomics, people have begun to pay attention to the role of protein molecules in animal reproduction. Although proteins participate in most physiological processes, little is reported about their role in sheep fertility. It has been reported that a low level of ribosome-related protein may be related to the high ovulation rate of Han sheep (Miao et al., 2016b). According to Zhang et al. (2019), some proteins regulate ovulation by directly or indirectly controlling the effects of GnRH on various metabolic factors. However, joint analysis of multi-omics and rigorous functional verification is practically significant to reveal the mechanism of multiple births in sheep. Although there is a particular research foundation on sheep fecundity, studies focusing on the variation of genes in the ovaries of ewes with multiple and singleton breeds before ovulation are rare. This present study will combine multiple omics studies to identify critical regulatory factors related to sheep fecundity.

The present study aimed to find more genes or proteins related to the fecundity of multiple births in sheep, which is helpful for sheep breeding. Therefore, ovarian samples of Small Tail Han sheep and Xinji fine wool sheep in the natural estrous cycle were collected and analyzed for the differentially expressed genes and proteins. After screening for the candidate genes, the overexpression and interference experiments were carried out on the genes in granulosa cells.

## Materials and Methods

### Ethical Statement

All sheep were normally reared and maintained in good health. Disturbance to the sheep was kept to the minimum, and suffering or injury was not inflicted on the animals during the experiment. All experiments were performed following the relevant animal experimentation guidelines and regulations in China. This study was approved by the Jilin Academy of Agricultural Sciences with approval No. 542256.

### Experimental Design

In this study, Small Tail Han sheep (multiparous, marked as Mult) and Xinji fine wool sheep (Chinese Merino, singleton, marked as Sgl) were used as multiparous and singleton bearing ewes, respectively. All the sheep were 5.5 years old ewes with three times lambing record. Only the ewes with more than three lambs per litter were selected for Small Tail Han sheep, and 12 healthy animals were included in each group. The animals were raised on the farm in Jilin Academy of Agricultural Sciences (sheep of the same breed were grouped). They were subjected to drinking and feeding *ad libitum*. The feeding, management and experiments on the animals were carried out according to the regulations approved by the Experimental Animals Committee of China Agricultural University.

### Serum Sample Collection and Hormone Determination

Ewes were checked daily for estrous behavior using vasectomized rams in the estrus season (August 2019, Changchun, China). The first estrous performance time of every ewe was accurately recorded as 0 day. During the estrus period of sheep (from 0 to 17.5 days), the blood of jugular vein for Mult and Sgl was collected using a coagulation promoting tube every morning and evening (3 ml each time, 7:00 a.m. and 19:00 p.m.). The blood tubes were left at room temperature for 30 min and then centrifuged at 1,500 × *g* for 10 min. Serum samples were collected and stored at −20°C. Enzyme-linked immune sorbent assay (ELISA) was carried out to measure the concentration of follicle-stimulating hormone (FSH), luteinizing hormone (LH), estradiol (E2) and progesterone (P4) in serum using an ELISA kit (BNIBT, Beijing, China), following the instructions of the manufacturer. Briefly, a sandwich ELISA was performed by adding standard dilutions or diluted samples to wells coated with antibodies. After washing, an enzyme-linked polyclonal antibody specific was added to the wells. After washing, an enzyme-substrate was added, and after 30 min, the OD at 450 nm was read using an ELISA plate reader.

### Ovarian Sample Collection and Evaluation

After routine ovariectomy at 17.5 days (pre-estrus in the next estrus cycle), the ovaries were collected after the first estrous performance time point. After ovariectomy, the development status of ovarian follicles was observed and recorded. Ovary samples of the sheep were washed with normal saline and snap frozen in liquid nitrogen. The ovary samples were divided into two groups: the Small Tail Han sheep group (Mult) and the Xinji fine wool sheep with large follicles group (Sgl). Each group contained three replicates, each having at least three ovaries from three different sheep. Furthermore, the follicle diameter and follicle number were also counted and recorded with three biological replicates and five technical replicates.

### Total RNA Extraction, Sequencing, and Candidate mRNA Identification

According to the instructions of the manufacturer, total RNA was extracted from ovary tissues of Mult and Sgl using TRIzol reagent (Takara, Shiga, Japan). After RNA purification, ribosomal RNA was removed from total RNA samples using a Ribo-zero rRNA Removal Kit (EPICENTRE, Madison, WI, United States). About 10 μg of total RNA from each mixed group was used to prepare libraries. Sequencing libraries were constructed using the NEB Next Ultra Directional RNA Library Prep Kit for Illumina (NEB, Ipswich, MA, United States). Briefly, the RNA was first converted to cDNA using random hexamer primer and M-MuLV Reverse Transcriptase and then cut into small bands of ∼380 bp using a Covaris DNA ultrasonic interrupter. Adaptors were ligated to the bands, and the libraries were prepared. Then all the libraries were sequenced on an Illumina HiSeq 2,500 platform with 125-base pair-end reads in Novogene Biotechnology Co., Ltd. (Novogene, Tianjin, China). For miRNA, Illumina’s TruSeq small RNA library preparation kit was used to prepare the miRNA library from samples. The sequence data were filtered to obtain clean reads by FastQC (v 0.11.4) with the default parameters. The clean data were assembled and compared with the reference genome of sheep (https://sheephapmap.org/news/OARv2p0) using HISAT2 (Kim et al., 2015). The value of FPKM (expected number of fragments per kb per million reads) of reads in each sample was calculated using Cufflinks (version 2.2.1). The differentially expressed genes (DEGs) by different comparisons were identified using DESeq2 (v1.6.3) (Love et al., 2014). Differential expression analysis was performed on the negative binomial distribution test and Benjamini–Hochberg method. An adjusted *p*-value (Q-value) <0.05 and |log2 fold change (FC)| >2 was considered as the thresholds for screening DE-mRNA, DE-miRNA, and DE-lncRNA significantly (Qian et al., 2017). Genes identified as log2FC >1 and log2FC <−1 were identified as up and downregulated DEGs, respectively. A heatmap was drawn on the DE-mRNA, DE-miRNA, and DE-lncRNA using the pheatmap R package (Kolde and Kolde, 2015).

### Proteome Sample Preparation, LC-MS/MS Measurements, and Data Processing

Ovary tissues were lysed in sodium deoxycholate (SDC) lysis buffer containing 4% (wt/vol) SDC and 100 mM Tris–HCl (pH 8.5). Proteome preparation was done using StageTip (iST) method (Kulak et al., 2014). Samples were separated using HPLC in a single run (without pre-fractionations) and analyzed with MS. The peptides were separated on a reverse-phase column, 50 cm packed in-house with 1.9-μm C18-Reprosil-AQ Pur reversed-phase beads (Dr Maisch GmbH, Ammerbuch, Germany) for about 120 min (single-run proteome analysis). Eluting peptides were electrosprayed and analyzed using tandem MS on a Q Exactive HF (Thermo Fischer Scientific) using higher-energy collisional dissociation (HCD)-based fragmentation, which was set to alternate between a full scan followed by up to five fragmentation scans. Raw MS data were analyzed with MaxQuant (version 1.5.1.6) (Cox and Mann, 2008) and using the Andromeda engine for database search. The MS/MS spectra were matched against the UniProt database (https://www.uniprot.org), with a false discovery rate (FDR) of <1% at the level of proteins, peptides, and modifications. The search results were then filtered using a cutoff of 1% for the peptide false identification rate. Label-free quantification was used to quantify the proteins. For quantitative changes, a cutoff of ≥1.5 or ≤0.666-fold change and Q-value <0.05 were set for differentially expressed proteins (Wei et al., 2018).

### Overexpression of the Target Gene in Ovarian Granulosa Cells

The pIRES2-ZsGreen1 vector sequence was purchased from Takara Bio (Takara, Shiga, Japan), and the structure of the vector is shown in Supplementary Figure S1. The plasmid was prepared by inserting cDNAs from the mRNA (*HSD17B1*) of interest and lncRNA (MSTRG.28645) into pIRES2-ZsGreen1 plasmid at Xho I and BamH I sites using restriction enzyme (TaKaRa, Otsu, Shiga, Japan) and T4 DNA ligase (NEW ENGLAND BioLabs, Ipswich, MA, United States). After identification and replication, the extracted plasmid was prepared for the transfection of granular cells. According to the instructions of the manufacturer, the transient transfection was achieved by Lipofectamine TM 3000 (Thermo Fisher Scientific, CA, United States) reagent. The siRNA oligonucleotides were synthesized and purified by Genomeditech Inc. (Shanghai, China), which also provided the scrambled negative control. According to the instructions of the manufacturer, granulosa cells (1 × 10^6^ cells/well) were grown to 80% confluence and transfected with 5 nM CD36 siRNA for 6 h using Lipofectamine 2000 (Life Technologies).

### MTT Assay and Flow Cytometry Analysis

The MTT assays were used to determine cell proliferation. The cells were seeded in a 96-well plate with a density of optimized cell number (5,000 cells/well). After 48 h of seeding, 20 µl of MTT (5 mg/ml) was added to the wells. Four hours later, the mixed medium was replaced with 150 µl of dimethyl sulfoxide (Sigma, St. Louis, MO, United States). The 96-well plate was then agitated for 15 min at room temperature. The OD value of each well was measured using a fluorescence microplate reader (Sunrise Remote, Tecan Austria GmbH, Grödig, Austria) at a wavelength of 490 nm. An AnnexinV-APC/7-AAD cell apoptosis detection kit (Nanjing Kaiji Biology, KGA1026) was used to detect cell apoptosis for cytometry analysis. The cells were briefly digested using trypsin and then washed using pre-cooled PBS. The binding buffer suspended cells were added to the flow tube along with 5 µl ANNEXIV-APC and 5 µl 7-AAD. The final solution was shielded from light exposure and left to react at room temperature for 5–15 min. Last, apoptosis was assessed using flow cytometry in each group.

### Quantitative Real-Time PCR Analysis

Detailed procedures for qRT-PCR were carried out as previously described by Zou et al. (2016). All samples were performed in triplicate, and the 2^−ΔΔCT^ calculation method was used to analyze the mRNA expressions after normalization with glyceraldehyde-3-phosphate dehydrogenase or actin housekeeping gene expressions, where necessary. The primers used in qRT-PCR are shown in Supplementary Table S1. qRT-PCR conditions were 95°C for 3 min followed by 40 cycles of 95°C for 15 s, 60°C for 40 s.

### Statistical Analysis

Statistical analysis was performed using SPSS statistical software (version 22.0). Statistical analysis was performed by using Student’s *t*-test to compare two groups and analysis of variance (ANOVA) to compare three or more groups. A value of *p* < 0.05 was considered statistically significant. The 2^−ΔΔCT^ method was used to determine the relative expression levels.

## Results

### Serum Hormone Indexes

**FIGURE 1:**
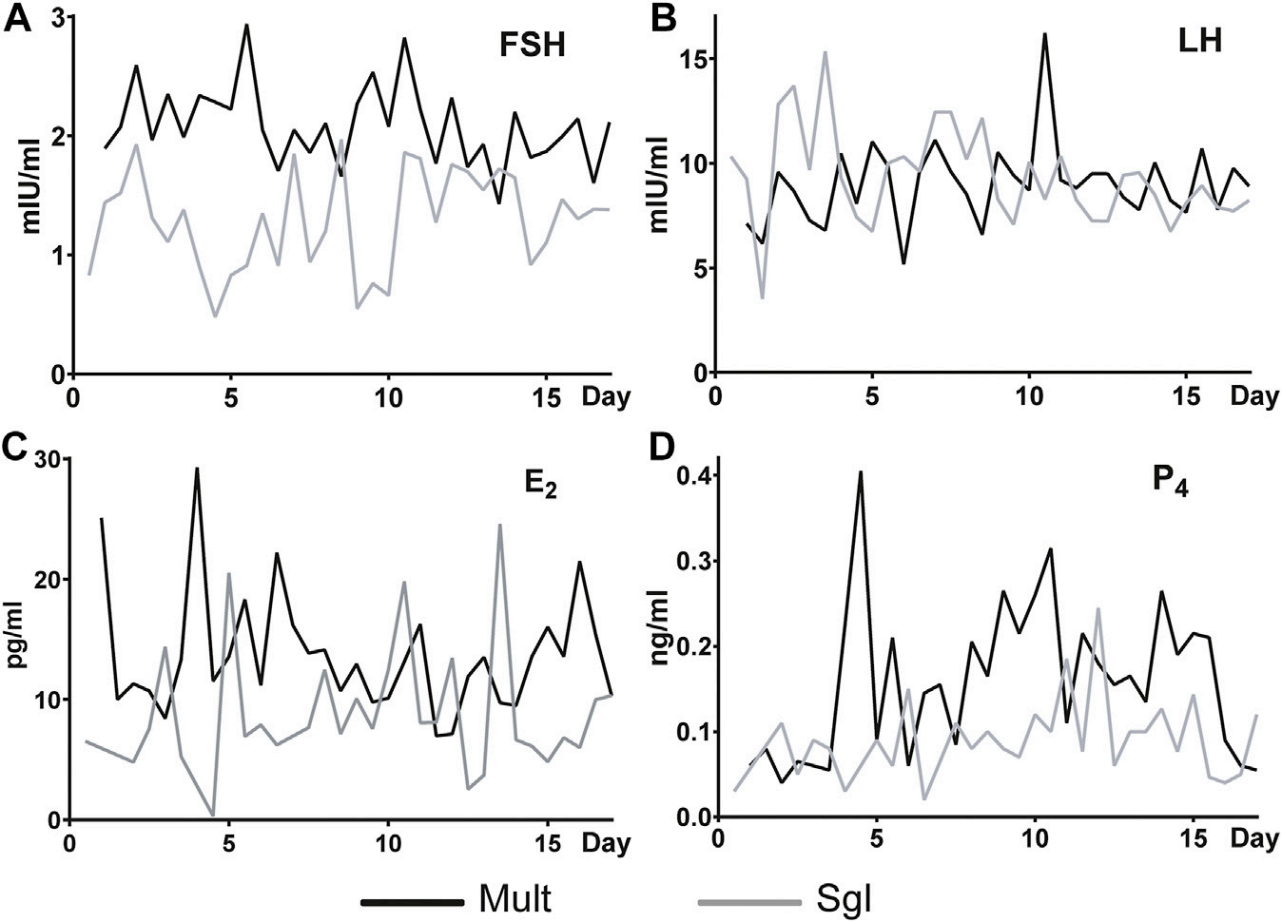
Serum FSH, LH, E2, and P4 levels in Small Tailed Han sheep and Xinji fine wool sheep. FSH, follicle-stimulating hormone; LH, luteinizing hormone; E2, estradiol; P4, progesterone. Mult, Small Tail Han sheep group, n = 12; Sgl, Xinji fine wool sheep group, n = 12. The abscissa is the number of days since the first estrus. Since the first estrus, blood samples were taken twice a day until 17.5 days (the next estrus round).

The concentration and trend of FSH, LH, E2, and P4 in serum was used to indicate the difference of the main reproductive hormones between the two breeds of sheep. Generally, it was found that the content of FSH in Mult was higher than that in Sgl sheep (Figure 1A). There was no significant difference in the content of LH between the two groups (Figure 1B). At most sampling time points, it was evident that Mult had a higher concentration of E2 than Sgl with the exception of 3, 5, 10, 12, and 13 days (Figure 1C). The content of P4 was found to be higher in Mult than in Sgl during 3–5, 8–11, and 12–17 days (Figure 1D). Furthermore, it was revealed that all these hormones were secreted in pulses. In summary, Mult had a higher average concentration of FSH, E_2,_ and P_4_ than Sgl.

### Ovarian Histomorphology

Rising hormone secretion levels may affect folliculogenesis and ovarian development. Therefore, the current study examined folliculogenesis between ovaries in Mult and Sgl. There were differences between ovaries in Mult and Sgl. Before ovulation, there was no significant difference in the number of small follicles (less than 3.5 mm in diameter) between Mult and Sgl groups (Table 1, *p* > 0.05). The number of large follicles (more than 3.5 mm in diameter) in the Mult group was more than that in the Sgl group (Table 1, *p* < 0.05). Nonetheless, there were large follicles on the ovaries of both sides. In the Sgl group, the ovary containing a large follicle was found only on one side of the ovary.

**TABLE 1:** Statistical results of the number of bilateral ovarian follicles.

Diameter	Mult (sum of bilateral ovaries)	Sgl (sum of bilateral ovaries)
<2 mm	7.2 ± 1.60	6.6 ± 1.36
2–3.5 mm	11.0 ±1.41	9.6 ± 1.96
>3.5 mm	3.8 ± 0.75 a	1.4 ± 0.49 b

Note. The letters indicate the results of analysis of variance, and different letters indicate significantly different values at *p* < 0.05.

### Transcriptome Analysis of Ovine Ovary

**FIGURE 2:**
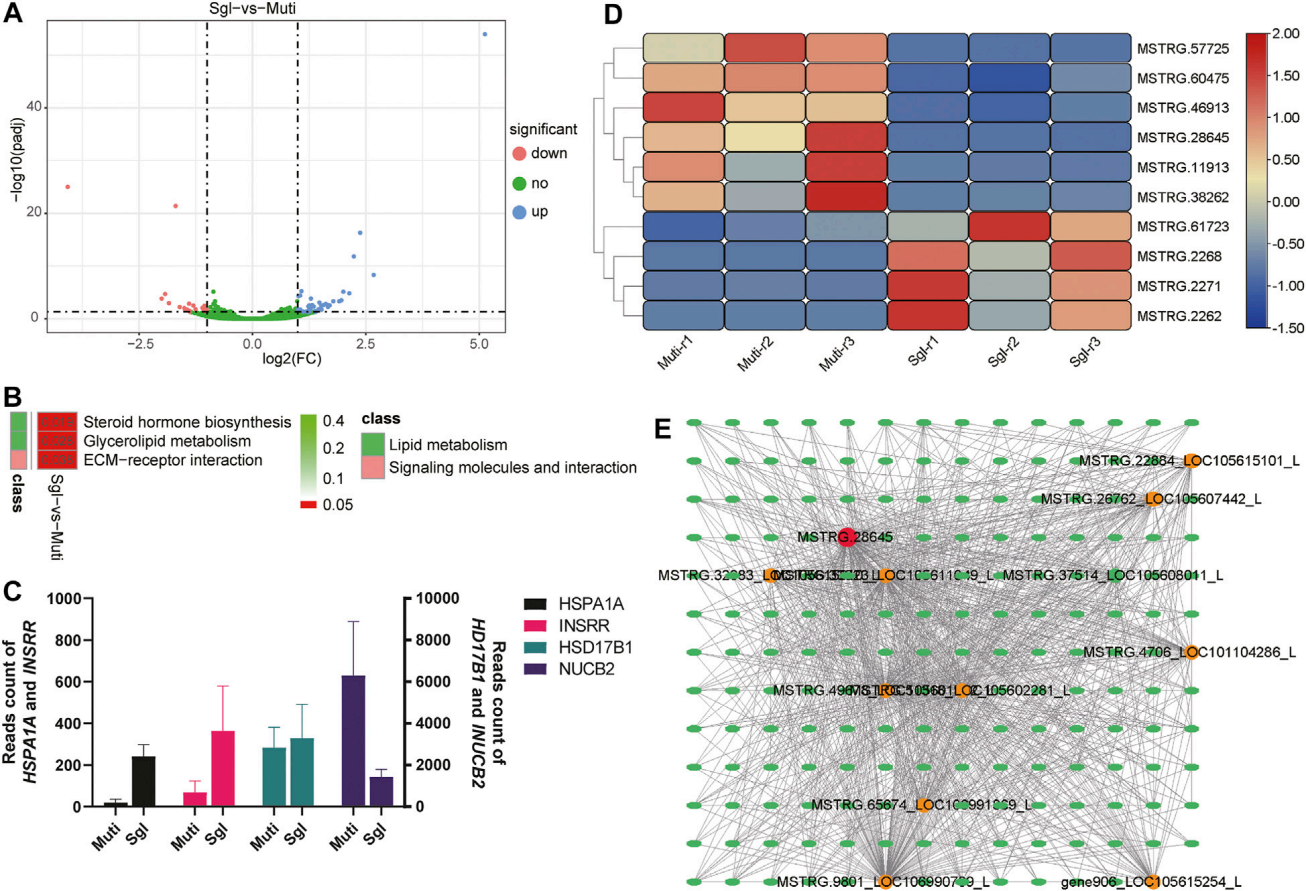
Transcriptome analysis results. Mult, Small Tail Han sheep group, n = 3; Sgl, Xinji fine wool sheep group, n = 3; DEGs, differentially expressed genes. **(A)** Volcano plot analysis of upregulated (the blue dots) and downregulated (the magenta dots) genes between Mult and Sgl. **(B)** The KEGG enrichment analysis of DEGs between Mult and Sgl. The redder the cell color, the smaller the *p*-value, and the number on the cell is the *p*-value. **(C)** The read count number of the four genes with the largest difference in expression levels. **(D)** The heatmap shows the expression levels of the 10 differently expressed lncRNAs (red, high expression; blue, low expression). **(E)** The interaction network of mRNAs and lncRNAs. Ellipses represent mRNA, and circles represent LncRNA. The redder the color of the circle, the more mRNA connected to the lncRNA.

Transcriptome analysis was conducted to delineate the genes and pathways difference in Mult and Sgl sheep. A total of 855.71 Mb reads were obtained from the nine ovarian samples. The summary of the RNA-seq reads for three cDNA libraries is listed in Supplementary Table S2. After filtering, a total of 62 canonical genes were identified as differentially expressed genes (DEGs) between Mult and Sgl sheep (Figure 2A). It was found that the DEGs were significantly enriched in steroid hormone biosynthesis (ko00140), ovarian steroidogenesis (ko04913), glycerolipid metabolism (ko00561), and ECM–receptor interaction (ko04512) (Figure 2B).

The details of DEGs and enriched pathways are listed in Supplementary Table S3. All the DEGs, *HSD17B1*, *HSPA1A*, *INSRR*, and *NUCB2* genes showed the largest differences in expression between the two groups (Figure 2C). It was evident that the *HSD17B1* gene was involved in both the steroid hormone biosynthesis and ovarian steroidogenesis pathways. Nonetheless, all the other genes involved in the two pathways did not show significant differences in their expression. This study hypothesized that the HSD17B1 gene is one of the genes that play an essential role in the biological processes related to sheep ovulation. The expression difference of *HSD17B1* between Mult and Sgl sheep may be related to litter size.

In addition, a total of 12 lncRNAs were identified in the current study. Of the 12 lncRNAs, 10 lncRNAs were significantly expressed in both Mult and Sgl (Figure 2D). Furthermore, to screen the significant lncRNAs, the interaction network between mRNAs and lncRNAs was analyzed based on the published criteria (Liao et al., 2011). As shown in Figure 2E, there were 1,118 sets of correlations between lncRNAs and mRNAs. It was found that *MSTRG.28645* was significantly related to 11 lncRNAs and 169 mRNAs in the co-expression network. Consequently, MSTRG.28645 should be profiled as a key lncRNA because it had the most links with other genes.

### Proteomics Analysis of Ovine Ovary

**FIGURE 3:**
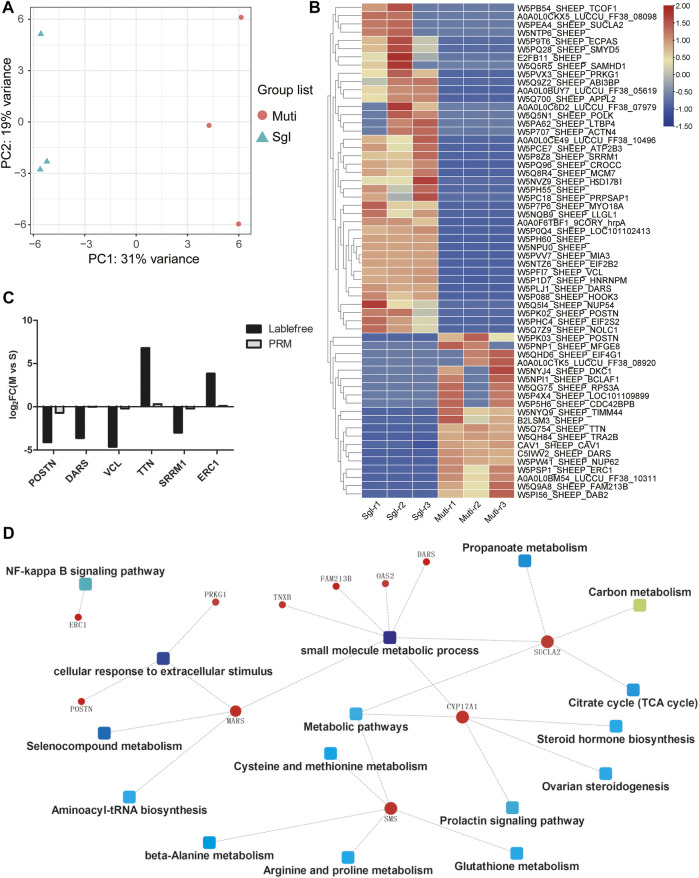
Proteomics analysis results. Mult, Small Tail Han sheep group, n = 3; Sgl, Xinji fine wool sheep group, n = 3; PRM, Parallel reaction monitoring. **(A)** Principal component analysis plots for all the samples. PC1, principal component 1; PC2, principal component 2. **(B)** The heatmap shows the levels of these 60 differentially expressed proteins (red, high expression; blue, low expression). **(C)** PRM verification of label-free differentially expressed protein expression. **(D)** Protein–protein interaction network showing the related signaling pathways.

Proteomic profiling revealed the significant differences between Mult and Sgl sheep. Of the approximately 2,100 proteins identified in each group, it was found that there were 2,031 known protein IDs. Principal component analysis (PCA) showed that the samples of the same group were clustered together (Figure 3A). There were 60 DEPs identified between Mult and Sgl sheep, 40 of which were more highly expressed in Sgl, whereas 20 DEPs were more expressed in Mult sheep (Figure 3B; Supplementary Table S4). Six DEPs (POSTN, DARS, VCL, TTN, SRRM1, and ERC1) were revalidated with the parallel reaction monitoring (PRM) method.

The results of this study showed that the protein expression trends detected with the two methods were consistent (Figure 3C). Furthermore, the protein–protein interaction network was constructed by enrichment of DEPs through signaling pathways and biological functions (Figure 3D). It was found that there were 11 proteins (POSTN, ERC1, DARS, PRKG1, MARS, SMS, TNXB, FAM213B, OAS2, SUCLA2, and CYP17A1) that interacted with each other through multiple signal pathways and biological functions. The CYP17A1 played a major role in multiple pathways as a hub protein. Therefore, both CYP17A1 protein and HSD17B1 gene were involved in the regulation of steroid hormone biosynthesis and ovarian steroidogenesis. This confirms that the two pathways play a crucial role in the litter size mechanism in sheep.

### Combined Proteome and Transcriptome Analysis

**FIGURE 4:**
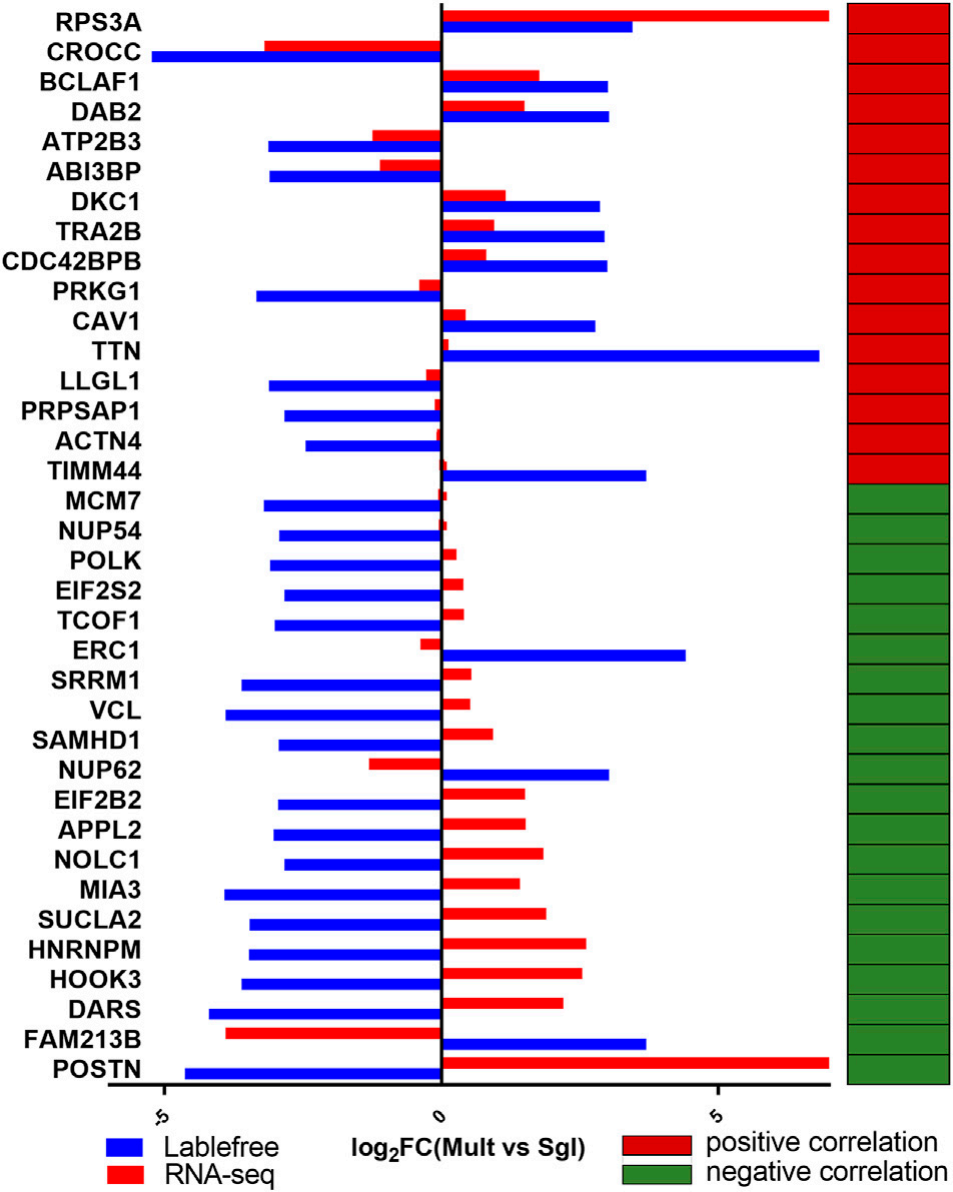
Analysis on the expression trend consistency of differentially expressed protein genes. Mult, Small Tail Han sheep group; Sgl, Xinji fine wool sheep group. The blue and red column diagrams represent the expression of protein and mRNA, respectively. The red and green cells show a positive and negative correlation, respectively, between the expression trend of the corresponding mRNA and proteome.

Among all the 62 DEGs identified from the canonical genes, the encoded proteins corresponding to 13 genes were detected using label-free quantification. The downregulation trend of *HSPA1A*, *NUCB2*, *CFH*, *RBM3*, *LDHA*, and *ACTN2* were opposite in transcriptome and proteome, while seven genes (HSD17B1, FKBP5, SERPINE1, KRT8, CRABP1, TOP1, and THY1) were consistent in the expression trend of transcriptome and proteome (Figure 4). Previously, HSD17B1 gene has been shown to participate in the reproductive regulation of mice and rats (Hakkarainen et al., 2015; Yuan et al., 2021). Therefore, in the current study, HSD17B1 was chosen as the key regulator for subsequent verification. Furthermore, the lncRNA MSTRG.28645 was also treated as a key lncRNA for the subsequent validation.

### Functional Verification of Candidate Genes in Granulosa Cells

**FIGURE 5:**
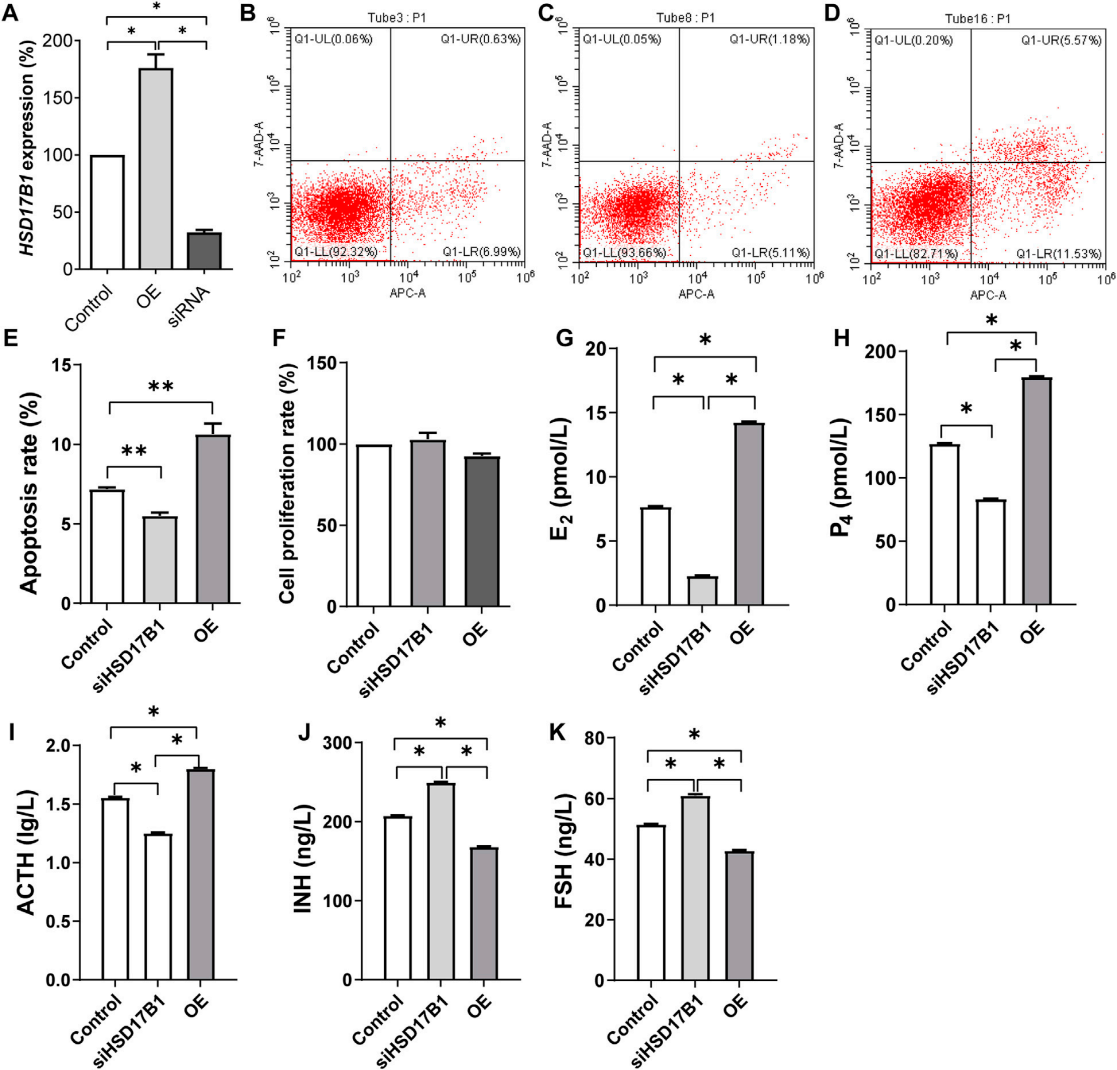
Functional verification of HSD17B1 gene in granulosa cells. E2, estradiol; P4, progesterone; ACTH, adrenocorticotropic hormone; INH, inhibin-B; FSH, follicle-stimulating hormone; OE, overexpression. *p < 0.05; **p < 0.01. The data are expressed as mean ± SD. **(A)** Relative expression of HSD17B1 in granulosa cells after over expression and interference treatment. **(B–D)** The results of flow cytometry apoptosis in control, siRNA interference group and overexpression group, respectively. **(E**, **F)** show the statistical results of apoptosis rate and cell proliferation rate, respectively. **(G–K)** The level of E2, P4, ACTH, INH, and FSH, respectively. Mult, Small Tail Han sheep (multiple births), n = 3; Sgl, Xinji Fine Wool sheep (singleton), n = 3.

Comprehensive gene expression and gene annotation finally selected *HSD17B1* as the candidate gene for the functional verification. As expected, the expression of HSD17B1 was upregulated and downregulated in the overexpressed and siRNA interfered granulosa cells, respectively (Figure 5A). The flow cytometry analysis results showed that the rate of apoptosis in granulosa cells for the *HSD17B1* interfered (5.50% ± 0.21%) and overexpressed cells (10.64% ± 0.66%) were significantly lower and higher than in the control group (7.18% ± 0.11%), respectively (Figures 5B–E). In addition, the MTT method was also performed to detect the proliferation rate of sheep granulosa cells after *HSD17B1* overexpression and interference treatment.

**FIGURE 6:**
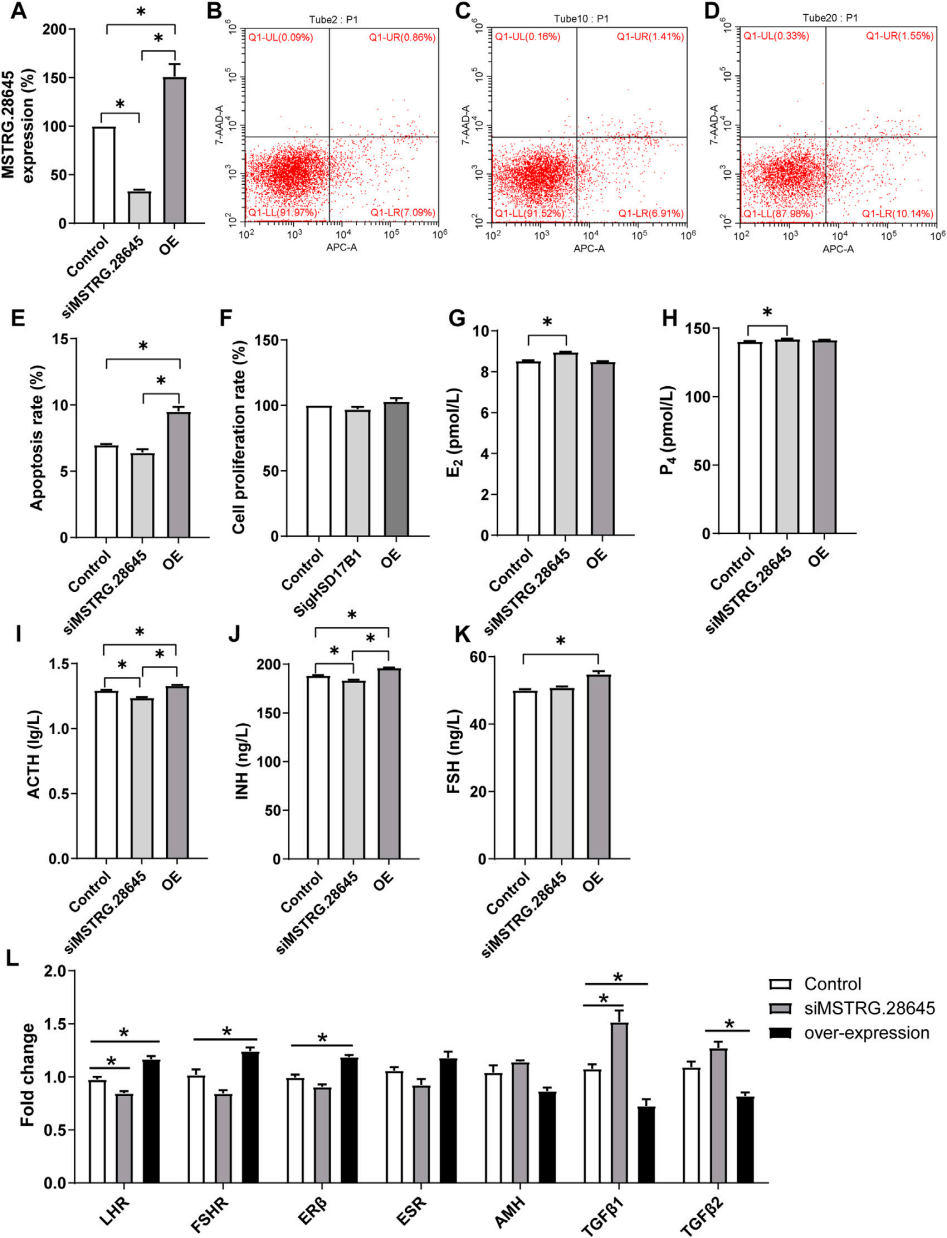
Functional verification of MSTRG.28645 in granulosa cells. E2, estradiol; P4, progesterone; ACTH, adrenocorticotropic hormone; INH, inhibin-B; FSH, follicle-stimulating hormone; OE, overexpression. *p < 0.05; **p < 0.01. The data are expressed as mean ± SD. **(A)**. Relative expression of MSTRG.28645 in granulosa cells after over expression and interference treatment. **(B–D)** The results of flow cytometry apoptosis in control, siRNA interference group, and overexpression group, respectively. **(E, F)** The statistical results of apoptosis rate and cell proliferation rate, respectively. **(G–K)** The level of E2, P4, ACTH, INH, and FSH, respectively. **(L)** Effect of MSTRG.28645 on gene expression in granulosa cells. Mult, Small Tail Han sheep (multiple births), n = 3; Sgl, Xinji Fine Wool sheep (singleton), n = 3.

Results of this study showed that there was no significant difference between interference, overexpression, and the control groups (Figure 5F). The E2, P4, ACT, INH, and FS levels were then detected in HSD17B1 interference and overexpression cells. The results showed that the *HSD17B1* gene could promote the secretion of E2, P4, and ACT, but inhibit the secretion of INH and FS (Figures 5G–K). By analyzing the location of MSTRG.28645 in the sheep genome, it was found that MSTRG.28645 was located on chromosome 6. The results of this study revealed that MSTRG.28645 can promote the apoptosis rate after successful overexpression and interference of MSTRG.28645 in granulosa cells (Figure 6A–E). In addition, it was evident that MSTRG.28645 also inhibits the secretion of E2 and P4 (Figures 6G–K). To further verify the role of MSTRG.28645, the expression of genes that were related to follicular development including LHR, FSHR, Erβ, ESR, and AMH were detected as well as the key genes in the TGF-β signaling pathway (TGFβ1 and TGFβ2). Moreover, it was found that SFR1 had a promoting effect on LHR, FSHR, and ERβ in sheep granulosa cells as well as an inhibitory effect on TGFβ1 and TGFβ2 (Figure 6L).

## Discussion

Ovaries are one of the essential organs in the animal reproductive system. The number of large follicles before ovulation reflects the level of ovarian development and ovulation potential (Hurley et al., 2020). It was evident that the diameter of large follicles before ovulation in Xinji fine-wool sheep is significantly larger than that in Small Tail Han sheep. That Small Tail Han sheep were found to have more large follicles (distributed on both ovaries) in the present study was consistent with the findings of Lazaridou et al. (2017). This study found that in the natural estrus cycle of sheep, the FSH, E2, and P4 levels in Small Tail Han sheep were significantly higher than those in Xinji fine-wool sheep. The length of time that FSH is maintained at a high level might be critical for Small Tail Han sheep to have multiple births. In addition to the role of P4 in inducing estrous behavior, it might also play a role in maintaining the development potential of the follicles.

Through transcriptomics analysis, it was evident that 61 DEGs were significantly enriched in signaling pathways like ECM–receptor interaction, focal adhesion, and PI3K–Akt. Furthermore, the expansion of cumulus–oocyte complexes (COCs) is essential for ovulation, and the extracellular matrix (ECM) is the basis of expansion of COCs (Dunning et al., 2014; Appeltant et al., 2015; Chen et al., 2016; Di Giacomo et al., 2016; Ferré et al., 2016). After detailed analysis, it was also found that most of the *HSD* gene family members were highly expressed in Small Tail Han sheep. The expression of *HSD17B1* in Small Tail Han sheep was significantly higher than that in the Xinji fine-wool sheep. As previously documented, *HSD17B1* and *HSD17B2* are important genes for estrogen synthesis (Järvensivu et al., 2015). However, *HSD17B1* has dual functions of estrogen activation and androgen inactivation (Järvensivu et al., 2015; He et al., 2016). Through overexpression and interference of the *HSD17B1* gene, it was found that the gene can promote E2, P4, and ACT secretion in sheep granulosa cells. It also has an inhibitory effect on the secretion of INH and FS, which is consistent with the findings of other previous studies (Hakkarainen et al., 2015; He et al., 2016). According to the present study, it was speculated that the lower expression of *HSD17B1* in Small Tail Han sheep decreases E2, P4, and ACT secretion and further reduce the apoptosis of granulosa cells, which could have maintained the development of follicles. The function of regulating these sex steroids of *HSD17B1* in granulosa cells had previously been reported in rats, mice, and horses (McGee and Hsueh, 2000; Gangloff et al., 2001; Zhang et al., 2012; Lazaridou et al., 2017). This study confirmed that *HSD17B1* might play a similar role in sheep.

Steroidogenesis and the expression of steroidogenesis-related genes in theca cells are primarily under the control of the LH/LHR pathway (Young and McNeilly, 2010). According to Korach et al., intrafollicular ERα inhibits androgen synthesis in theca cells by repressing CYP17A1 expression (Taniguchi et al., 2007). Elsewhere, Imamichi et al. found that mice that expressed increased levels of CYP17A1 secrete significantly higher amounts of androgens (Imamichi et al., 2017). To explain this finding, the current study speculated that the high expression of CYP17A1 in group Sgl might have accelerated the production of androgen and antagonized estrogen. In conclusion, the current study found that both HSD17B1 and CYP17A1 played a key role in regulating hormone secretion, resulting in the early termination of ovulation in singleton ewes, whereas the Small Tail Han sheep maintained a high level of estrogen and continued to promote ovulation. However, there is a need for further animal cloning experiments to verify this conclusion.

In the present study, the candidate lncRNA MSTRG.28645 showed a clear correlation with hormone secretion. In addition, the co-expression analysis of mRNA and lncRNA showed that MSTRG.28645 was an important hub gene. Furthermore, MSTRG.28645 is located on chromosome 6, on which the known fecundity gene *Fecb* is also located. After overexpression and interference experiments, it was found that MSTRG.28645 can significantly affect the secretion of estrogen. It was evident that the overexpression of MSTRG.28645 increased the abundance of *LHR*, *FSHR*, and *ERβ*, but decreased the TGFβ1 and TGFβ2.

Some previous investigations have indicated that prolific sheep and goats have higher FSHR expression in ovaries. It has also been reported that the level of FSHR mRNA in developing follicular cells is higher in polyembryonic breeds than in single-birth breeds. This finding implies that the greater ovulation rate in these breeds is associated with greater gonadotropin responsiveness during the early follicular phase (Abdennebi et al., 1999; Cui et al., 2009; Zi et al., 2013). The enhancement results of *LHR* and *FSHR* in MSTRG.28645 overexpressed cells in the present study indicated the potential role of MSTRG.28645 in promoting ovulation. Therefore, it is inferred that MSTRG.28645 is also one of the key lncRNA related to fecundity.

## Conclusion

There were significantly different expression levels of mRNA *HSD17B1* and lncRNA MSTRG.28645 in the ovaries of Small Tail Han and Xinji fine wool sheep, and both *HSD17B1* and MSTRG.28645 play a crucial role in hormone secretion in the granulosa cells, hence, affecting the fecundity of the sheep.

## Data Availability

The data that support the findings of this study are available from BIGSUB database (https://ngdc.cncb.ac.cn/gsub/) with project number PRJCA005970.
