# Supplementary figures and images for: Genome-Wide Identification of mRNAs, lncRNAs, and Proteins, and Their Relationship With Sheep Fecundity
Source: Front Genet. 2022 Feb 8;12:750947. doi: 10.3389/fgene.2021.750947 (PMC8861438; doi:10.3389/fgene.2021.750947)

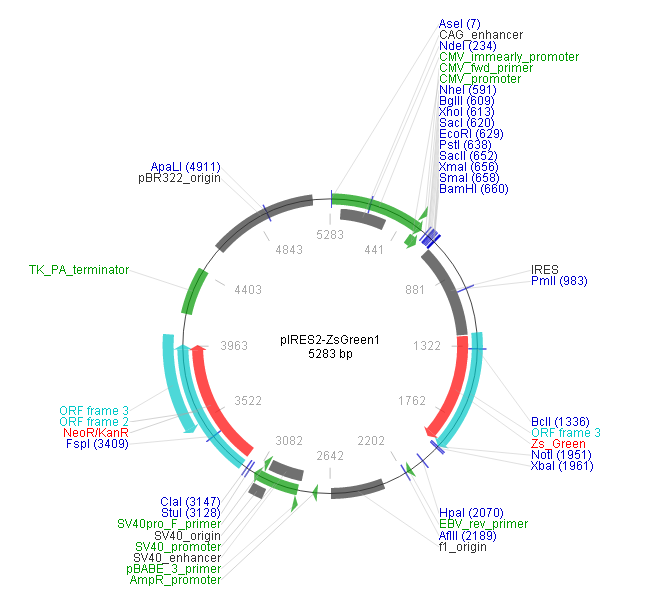

Supplement: Supplementary file 3 [file Image1.TIF]
